# Comparison of Percutaneous Kyphoplasty With or Without Posterior Pedicle Screw Fixation on Spinal Sagittal Balance in Elderly Patients With Severe Osteoporotic Vertebral Compression Fracture: A Retrospective Study

**DOI:** 10.3389/fsurg.2022.800664

**Published:** 2022-02-18

**Authors:** Quan Zhou, Junxin Zhang, Hao Liu, Wei He, Lei Deng, Xinfeng Zhou, Huilin Yang, Tao Liu

**Affiliations:** Department of Orthopaedics, The First Affiliated Hospital of Soochow University, Suzhou, China

**Keywords:** osteoporosis, vertebral compression fracture, percutaneous kyphoplasty, posterior pedicle screw fixation, spinal sagittal balance

## Abstract

**Objective:**

To compare the effects of percutaneous kyphoplasty (PKP) with or without posterior pedicle screw fixation (PPSF) on spinal sagittal balance in elderly patients with severe osteoporotic vertebral compression fracture (sOVCF).

**Methods:**

From January 2016 to December 2018, 102 elderly patients with single-level thoracolumbar sOVCF were enrolled. Among them, 78 cases underwent PKP (Group A), and 24 cases underwent PPSF+KP (Group B). Clinical evaluation included perioperative parameters, Oswestry Disability Index (ODI) and Visual Analog Scale (VAS) for back pain; Radiographic evaluation included anterior vertebral height (AVH) and rate (AVHr), local kyphotic angle (LKA), and spino-pelvic sagittal balance parameters.

**Results:**

Perioperative parameters including operation time, blood loss, fluoroscopic time and hospital stay in Group A were less than those in Group B (*p* < 0.05). Compared with the pre-operative results, the ODI and VAS scores of both groups decreased significantly in the three follow-ups after surgery (*p* < 0.05). The post-operative ODI and VAS scores of Group A were significantly better than those of Group B, but the results were opposite at the final follow-up (*p* < 0.05). Compared with the pre-operative values, except that there was no significant difference in pelvic incidence (PI) (*p* > 0.05), other radiographic parameters of both groups were improved significantly in the three follow-ups after surgery (*p* < 0.05). The AVH, AVHr, LKA and lumbar lordosis (LL) in Group B were better than those in Group A in the three follow-ups after surgery (*p* < 0.05). At the final follow-up, the sacral slope (SS) and pelvic tilt (PT) differed significantly between the two groups (*p* < 0.05).

**Conclusions:**

Both PPSF+KP and PKP can achieve favorable clinical outcomes and maintain the spinal sagittal balance. Compared with PPSF+KP, PKP showed more significant advantages in the early post-operative period. However, in the long-term follow-up, PPSF+KP showed better clinical outcomes and may be better than PKP in maintaining spinal sagittal balance.

## Introduction

Spinal sagittal balance is a good state for an individual to maintain the body in a stable position, which plays a crucial role in maintaining the normal biomechanics and physiologic function of the spine (1). When the spinal deformity gradually deteriorates and exceeds the overall compensatory capacity, it is no longer effective to maintain body balance by increasing muscle strength, resulting in the spinal sagittal imbalance. Some researchers have reported that correction of spinal sagittal imbalance is associated with favorable clinical efficacy after lumbar surgery (2, 3). Many spinal diseases, such as spinal deformity, lumbar spondylolisthesis etc., can lead to spinal sagittal imbalance (1, 4, 5). However, the spinal sagittal imbalance caused by osteoporotic vertebral compression fracture (OVCF) has not received enough attention.

OVCF is a fragile fracture caused by osteoporosis under the action of slight external force or not, causing intractable pain, lowering the quality of life, and also increasing the incidence of systemic complications and mortality (6–8). Percutaneous kyphoplasty (PKP) is one of the most widely used surgical methods for OVCF. This minimally invasive technique can achieve some benefits on short-term prognosis by eliminating pain and restoring vertebral height immediately after surgery (9). Although these advantages have been demonstrated, PKP is associated with a high risk of recollapse of fractured vertebrae or fractures in adjacent segments (10, 11). In particular, for patients with severe OVCF (sOVCF), defined as an expected reduction of two-thirds or more in anterior vertebral height (12), PKP alone may not be able to effectively correct severe kyphosis and maintain spinal sagittal balance in the long term, which may also increase the risk of adjacent segment fractures and vertebral recollapse. In addition, pedicle screws show the high biomechanical strength offered by three-column fixation, which can keep the vertebral stable and correct kyphosis to a certain extent. However, if only pedicle screw fixation is used in these patients, there would be a high risk of screw loosening, and late kyphosis deformity due to osteoporosis (13, 14). Therefore, to more effectively reduce the risk of adjacent vertebral fractures, correct kyphosis and maintain spinal sagittal balance, posterior pedicle screw fixation combined with kyphoplasty (PPSF+KP) has been used in recent years.

Some clinical studies have reported that PPSF combined with KP or vertebroplasty (VP) could be a good choice for patients with thoracolumbar OVCF, which can reduce the incidence of vertebral refractures and restore the height of the fractured vertebrae (15–17). So far, however, few studies have compared the prognosis of PKP and PPSF+KP in patients with thoracolumbar sOVCF, especially the long-term effect on spinal sagittal balance. Therefore, this retrospective comparative study was conducted to compare the effects of PKP and PPSF+KP on clinical function and radiographic outcomes in elderly patients with single-level thoracolumbar sOVCF.

## Data and Methods

### Selection Criteria

Inclusion criteria: (1) patients with a single-level thoracolumbar compression fracture (T11–L2); (2) patients with osteoporosis (T < −2.5) on dual energy X-ray absorptiometry (DEXA); (3) patients with sOVCF, defined as an expected reduction of two-thirds or more in anterior vertebral height (AVH); (4) patients with obvious back pain but without symptoms of nerve damage; (5) patients treated with PKP or PPSF+KP; (6) patients over 60 years of age. Exclusion criteria: (1) patients with previous fractures or surgical intervention at the spinal alignment; (2) fractures with tumor, tuberculosis or ankylosing spondylitis; (3) patients who died or were unable to complete 24 months of follow-up.

### General Information

According to the inclusion and exclusion criteria, a total of 102 elderly patients with sOVCF from January 2016 to December 2018 were enrolled in this retrospective study. Among them, 78 cases (Group A) received PKP, 24 cases (Group B) received PPSF+KP. All patients' data and imaging materials were obtained from the electronic medical record management system of our hospital. This study was carried out with the approval of our institution's ethics committee.

### Surgical Procedure

All patients were operated under general anesthesia. After anesthesia, they were placed in a prone position with the pelvis and manubrium supported by pads. The use of C-arm radiographs facilitated the acquisition of a standard anteroposterior and lateral images of the surgical vertebrae.

**Figure 1:**
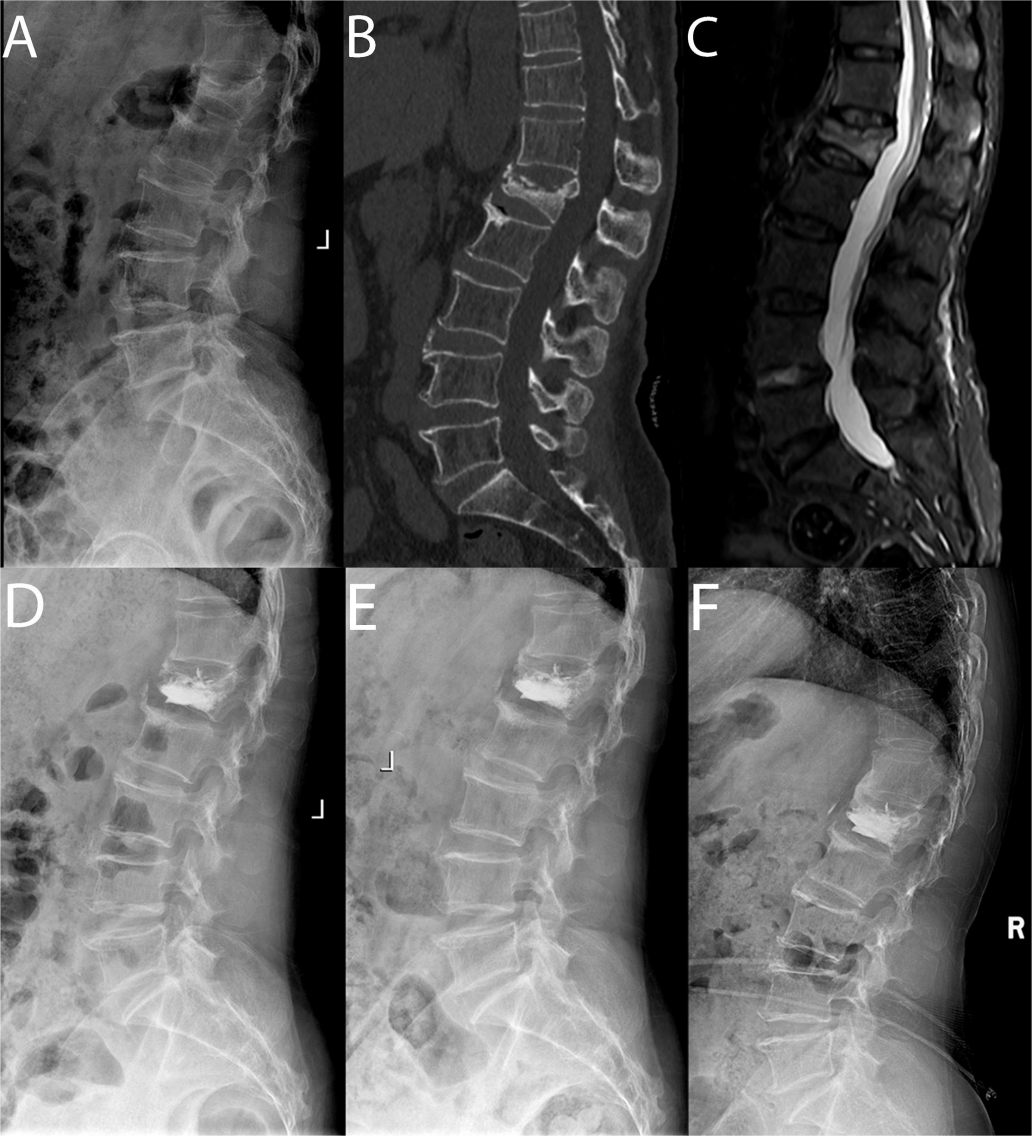
Pre-operative sagittal lateral view **(A)**, sagittal computed tomographic scan **(B)**, sagittal fat-suppressed sequence in MRI **(C)**, post-operative sagittal lateral view **(D)**, sagittal lateral view 1 month after surgery **(E)** and sagittal lateral view at the final follow-up **(F)** of a 64-year-old female patient with L1 sOVCF was treated with PKP.

For Group A, bilateral transpedicular working channels were penetrated into the surgical vertebrae by the cannula and trocar systems under fluoroscopic guidance. Then, each balloon was placed into the cavity of the intravertebral cleft in the surgical vertebrae through the working channel and inflated to over 150 psi. Polymethyl methacrylate (PMMA) and non-ionic contrast medium were prepared at 26 g/10 ml and injected carefully into the vertebrae using a bone cement injector under fluoroscopic monitoring. The incremental temperature cement delivery and graded infusion techniques were used in our hospital to minimize the leakage rate (18) (Figure 1).

**Figure 2:**
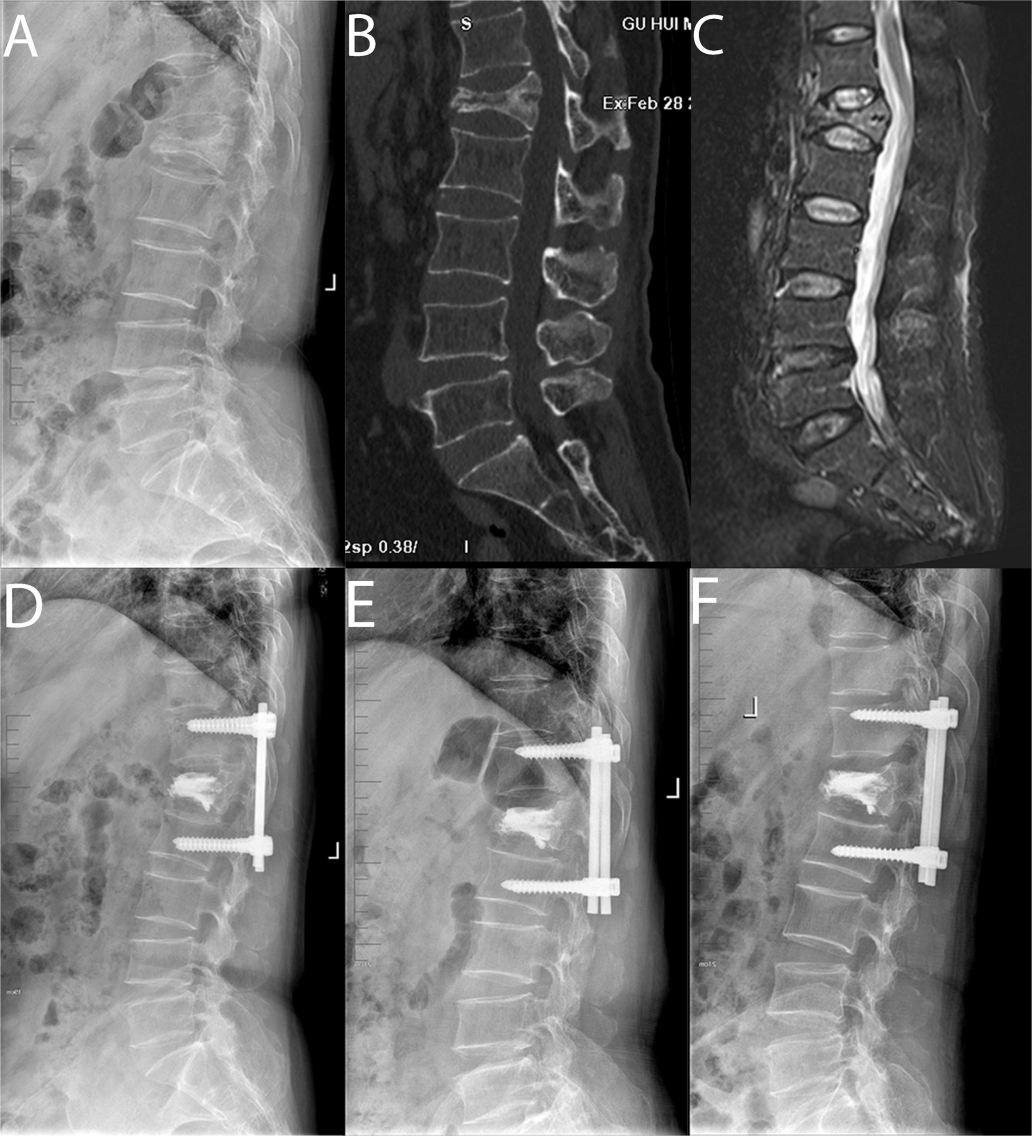
Pre-operative sagittal lateral view **(A)**, sagittal computed tomographic scan **(B)**, sagittal fat-suppressed sequence in MRI **(C)**, post-operative sagittal lateral view **(D)**, sagittal lateral view 1 month after surgery **(E)** and sagittal lateral view at the final follow-up **(F)** of a 62-year-old male patient with L1 sOVCF was treated with PPSF+KP.

For Group B, a standard open posterior midline approach was performed, centering the fractured vertebrae and systematically revealing the posterior vertebral structure. Under fluoroscopic monitoring, 4 pedicle screws were inserted into the adjacent upper and lower vertebrae of the surgical vertebrae, and the height of the fractured vertebrae was restored by position combined with internal fixation distraction and lateral lifting. In the second surgical phase, the procedure for PKP described above was used (Figure 2).

During the follow-up period, all patients performed functional exercise of the back muscles and took anti-osteoporosis drugs under the guidance of doctors.

### Clinical Evaluation

For the measurement of clinical outcomes, perioperative parameters, including operative time, blood loss, fluoroscopic time, cement volume and hospital stay, were recorded and all patients filled out the following questionnaires pre-operatively, post-operatively, 1 month after surgery and at the final follow-up: Oswestry Disability Index (ODI), and Visual Analog Scale (VAS) for back pain. The ODI scores were used to assess patients' improvement in quality of life, the VAS scores were used to evaluate patients' subjective pain perception (0–10 score, 0 indicated no pain, 10 indicated the most severe pain) (19).

### Radiographic Evaluation

**Figure 3:**
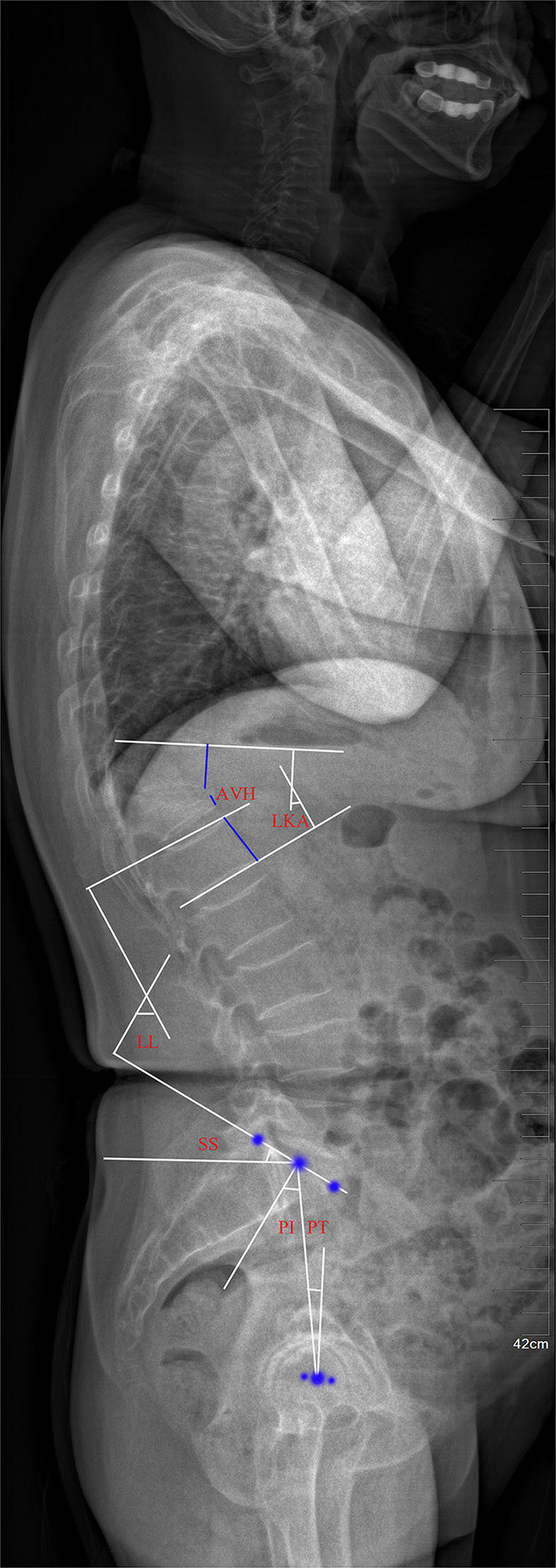
Plain lateral radiograph for measuring radiographic parameters. AVH, anterior vertebral height; LKA, local kyphotic angle; LL, lumbar lordosis; SS, sacral slope; PI, pelvic incidence; PT, pelvic tilt.

Bone mineral density (BMD) was assessed as T score in the lumbar spine with DEXA (Discovery Wi, Hologic, America). The anteroposterior and lateral radiographs in the standing position were routinely performed pre-operatively, post-operatively, 1 month after surgery, and at the final follow-up. The anterior height of the fractured vertebrae was measured, and the anterior vertebral height rate (AVHr) was calculated as a percentage of the average adjacent upper and lower vertebral height. The local kyphotic angle (LKA) was measured as the angle between the superior endplate of the vertebrae above and the inferior endplate of the vertebrae below the fractured level. The following parameters of spino-pelvic sagittal balance were measured (20): Lumbar lordosis (LL) was defined by Cobb's method as the angle between the superior endplate of L1 vertebrae and the sacral plate; sacral slope (SS) was defined as the angle formed between the sacral plate and the horizontal line; pelvic incidence (PI) was formed by the line perpendicular to the midpoint of the sacral plate and the line between the midpoint of the sacral plate and the centroid of femoral heads; pelvic tilt (PT) was formed by the angle between the line connecting the midpoint of the sacral plate with the centroid of femoral heads and the vertical line (Figure 3).

### Statistical Methods

SPSS 26.0 statistical software (SPSS Inc., Chicago, IL) was used for data processing. The measurement data were expressed as mean ± standard deviation ($\bar{x}$ ± s). Paired sample *T*-test was used for comparison in the same group. χ^2^ test was used for categorical variable data. *P* < 0.05 was considered statistically significant.

## Results

### Demographics

Demographic data of both groups were shown in Table 1. Among the patients included in this study, the average age was 66.12 ± 5.21 years old, male patients (16.67%) were less than female patients (83.33%), and most patients (60.78%) developed sOVCF after slight trauma. In terms of the fractured segment, L1 (46.08%) was the most common compared with other segments. There were no significant differences between the two groups in terms of age, gender, trauma history and fractured segments (*p* > 0.05). The mean body mass index (BMI) of Group A was slightly higher than that of Group B, but the difference was not statistically significant (*p* > 0.05). The mean BMD of all patients were −3.15 ± 0.41, and there was no significant difference between the two groups (*p* > 0.05). In terms of comorbidities, there were different numbers of patients with hypertension, diabetes, hyperlipidemia and smoking in both groups, but there was no significant difference between the two groups (*p* > 0.05). The average follow-up duration of all patients was 34.83 ± 5.90 months, and there was no significant difference between the two groups (*p* > 0.05).

**Table 1:** Demographic data of both groups.

	**Full sample**	**Group A**	**Group B**	***P*-value**
Number of patients	102	78	24	
Age (years)	66.12 ± 5.21	65.82 ± 5.21	67.08 ± 5.22	0.302
Gender (male/female)	17/85	14/64	3/21	0.531
Trauma history (*n*)				0.891
None	26 (25.49%)	20 (25.64%)	6 (25.00%)	
Slight	62 (60.78%)	48 (61.54%)	14 (58.33%)	
Severe	14 (13.73%)	10 (12.82%)	4 (16.67%)	
Fractured segment (*n*)				0.903
T11	9 (8.82%)	7 (8.97%)	2 (8.33%)	
T12	19 (18.63%)	14 (17.95%)	5 (20.83%)	
L1	47 (46.08%)	35 (44.87%)	12 (50%)	
L2	27 (26.47%)	22 (28.21%)	5 (20.83%)	
BMI (kg/m^2^)	24.74 ± 3.68	25.05 ± 3.64	23.74 ± 3.70	0.128
BMD (T-score)	−3.15 ± 0.41	−3.17 ± 0.37	−3.07 ± 0.52	0.286
Comorbidity (*n*)
Hypertension	44 (43.14%)	33 (42.31%)	11 (45.83%)	0.760
Diabetes	37 (36.27%)	26 (33.33%)	9 (37.50%)	0.707
Hyperlipidemia	53 (51.96%)	40 (51.28%)	13 (54.17%)	0.805
Smoking	21 (20.59%)	16 (20.51%)	5 (20.83%)	0.973
Follow-up (months)	34.83 ± 5.90	34.42 ± 6.06	36.17 ± 5.21	0.207

*BMI, body mass index; BMD, bone mineral density*.

### Clinical Outcomes

Perioperative parameters of both groups were shown in Table 2. Operation time, blood loss, fluoroscopic time, and hospital stay in Group A were all less than those in Group B (*p* < 0.05). In terms of injection volume of bone cement, Group A was slightly more than Group B, but there was no statistical difference (*p* > 0.05).

**Table 2:** Perioperative parameters of both groups.

	**Group A (*n* = 78)**	**Group B (*n* = 24)**	***P*-Value**
Operative time (min)	44.12 ± 7.40	116.04 ± 17.94	<0.001^*^
Blood loss (ml)	10.64 ± 4.72	60.63 ± 14.69	<0.001^*^
Fluoroscopic time (s)	39.19 ± 8.42	64.71 ± 8.99	<0.001^*^
Cement volume (ml)	6.43 ± 0.69	6.32 ± 0.67	0.483
Hospital stay (days)	4.93 ± 1.72	7.67 ± 2.10	<0.001^*^

* *Significance between the two groups, P < 0.05*.

**Figure 4:**
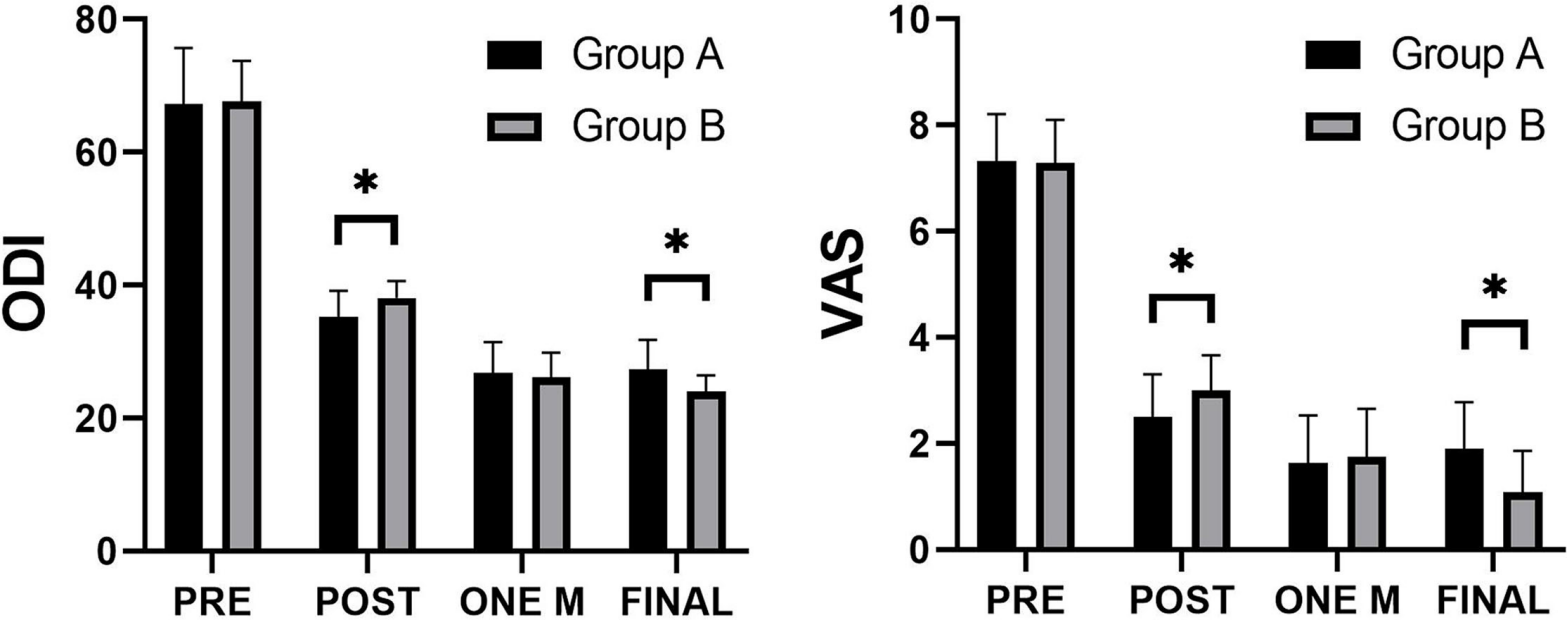
Comparison of the Visual Analog Scale (VAS) and Oswestry disability index (ODI) scores between the two groups. *Significance between the two groups, *P* < 0.05.

The ODI and VAS scores of both groups were shown in Figure 4. Compared with pre-operative results, the ODI and VAS scores of both groups post-operatively, one month after surgery and at the final follow-up all decreased significantly (*p* < 0.05). In addition, the ODI and VAS scores of Group A were significantly better than those of Group B post-operatively (*p* < 0.05), but the ODI and VAS scores of Group B were significantly better than those of Group A at the final follow-up (*p* < 0.05).

### Radiographic Outcomes

**Figure 5:**
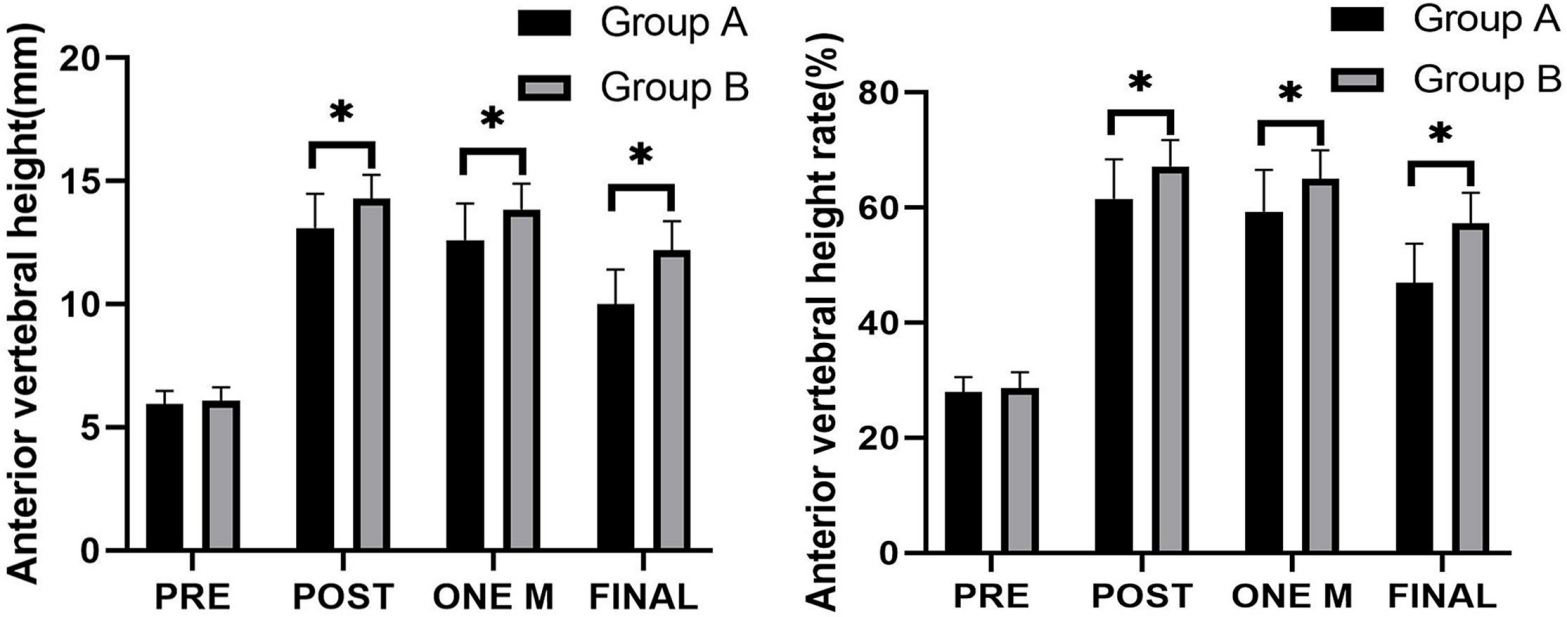
Comparison of the anterior vertebral height (AVH) and anterior vertebral height rate (AVHr) between the two groups. *Significance between the two groups, *P* < 0.05.

The AVH and AVHr of both groups were shown in Figure 5. Compared with the pre-operative results, the AVH and AVHr were all significantly increased in both groups post-operatively, 1 month after surgery and at the final follow-up (*p* < 0.05). In addition, the recoveries of AVH and AVHr in Group A were significantly better than those in Group B post-operatively, 1 month after surgery and at the final follow-up (*p* < 0.05).

The LKA of both groups was shown in Table 3. From T11 to L2, the LKA decreased gradually. Compared with pre-operative results, LKA of the fractured vertebrae decreased significantly in both groups post-operatively, 1 month after surgery and at the final follow-up (*p* < 0.05). In addition, the recovery of LKA in Group A was significantly better than that in Group B post-operatively, 1 month after surgery and at the final follow-up (*p* < 0.05).

**Table 3:** Local kyphotic angle of fractured vertebrae of both groups.

	**Group A (*n* = 78)**	**Group B (*n* = 24)**	***P*-value**
PRE (°)
T11 (*n* = 9)	27.43 ± 1.28	27.50 ± 0.50	0.527
T12 (*n* = 19)	24.93 ± 1.61	24.80 ± 1.70	
L1 (*n* = 47)	20.89 ± 5.57	20.00 ± 2.18	
L2 (*n* = 27)	17.95 ± 4.05	16.20 ± 0.70	
Series	21.37 ± 3.61	20.83 ± 3.71	
POST (°)
T11 (*n* = 9)	19.86 ± 1.81	17.50 ± 0.50	0.029^**^
T12 (*n* = 19)	17.50 ± 1.04	16.00 ± 3.50	
L1 (*n* = 47)	12.97 ± 4.26	11.67 ± 1.15	
L2 (*n* = 27)	12.27 ± 1.06	9.80 ± 0.70	
Series	14.21 ± 3.02^*^	12.67 ± 2.84^*^	
ONE M (°)
T11 (*n* = 9)	20.14 ± 2.14	18.00 ± 0.00	0.046^**^
T12 (*n* = 19)	17.79 ± 1.57	16.20 ± 2.70	
L1 (*n* = 47)	13.20 ± 4.87	12.00 ± 1.45	
L2 (*n* = 27)	12.68 ± 1.37	10.60 ± 0.80	
Series	14.50 ± 3.09^*^	13.08 ± 2.73^*^	
FIANL (°)			
T11 (*n* = 9)	21.43 ± 2.29	20.00 ± 2.00	0.029^**^
T12 (*n* = 19)	19.79 ± 2.49	17.40 ± 2.80	
L1 (*n* = 47)	15.03 ± 3.97	14.00 ± 2.00	
L2 (*n* = 27)	14.50 ± 2.83	11.80 ± 2.20	
Series	16.31 ± 3.06^*^	14.75 ± 2.83^*^	

*PRE, pre-operative; POST, post-operative; ONE M, one month after surgery; FINAL, final follow-up*.

* *Significance compared with the pre-operative, P < 0.05*.

** *Significance between the two groups, P < 0.05*.

Spino-pelvic sagittal balance parameters of both groups were shown in Table 4. Except that PI of both groups were not statistically different from the pre-operative results, there were significant differences in other parameters post-operatively, 1 month after surgery and at the final follow-up compared with the pre-operative results (*p* < 0.05). In addition, the maintenance of LL in Group A was significantly better than that in Group B post-operatively, 1 month after surgery and at the final follow-up (*p* < 0.05). At the final follow-up, SS and PT differed significantly between the two groups (*p* < 0.05). In terms of PI and PI-LL at the final follow-up, although the values of Group A were slightly higher than those of Group B, they did not reach significant differences (*p* > 0.05).

**Table 4:** Spino-pelvic sagittal balance parameters of both groups.

	**Group L (*n* = 78)**	**Group S (*n* = 24)**	***P*-Value**
LL (°)
PRE	38.27 ± 2.73	38.88 ± 3.42	0.374
POST	42.32 ± 2.44^*^	44.17 ± 2.18^*^	0.001^**^
ONE M	42.03 ± 2.72^*^	43.79 ± 2.50^*^	0.006^**^
FINAL	41.32 ± 2.38^*^	42.75 ± 3.42^*^	0.023^**^
SS (°)
PRE	32.63 ± 3.00	32.87 ± 3.58	0.737
POST	36.49 ± 3.04^*^	37.71 ± 3.56^*^	0.101
ONE M	36.31 ± 3.10^*^	37.58 ± 3.75^*^	0.097
FINAL	35.14 ± 2.54^*^	36.71 ± 3.74^*^	0.021^**^
PT (°)
PRE	21.47 ± 4.03	21.58 ± 3.46	0.905
POST	17.01 ± 3.50^*^	15.96 ± 3.16^*^	0.190
ONE M	17.14 ± 3.50^*^	16.17 ± 3.25^*^	0.229
FINAL	19.18 ± 3.99^*^	17.21 ± 3.30^*^	0.030^**^
PI (°)
PRE	54.10 ± 4.58	54.46 ± 4.39	0.738
POST	53.50 ± 3.72	53.67 ± 3.85	0.849
ONE M	53.45 ± 3.66	53.75 ± 4.00	0.731
FINAL	54.32 ± 4.36	53.92 ± 4.40	0.693
PI-LL (°)
PRE	15.83 ± 5.54	15.58 ± 5.56	0.847
POST	11.18 ± 4.59^*^	9.50 ± 4.24^*^	0.114
ONE M	11.42 ± 4.74^*^	9.96 ± 4.61^*^	0.186
FINAL	13.00 ± 5.13^*^	11.17 ± 5.56^*^	0.137

*LL, lumbar lordosis; SS, sacral slope; PI, pelvic incidence; PT, pelvic tilt; PRE, pre-operative; POST, post-operative; ONE M, one month after surgery; FINAL, final follow-up*.

* *Significance compared with the pre-operative, P < 0.05*.

** *Significance between the two groups, P < 0.05*.

### Related Complications

In terms of related complications, there were 10 cases (12.82%) in Group A and 2 cases (8.33%) in Group B, with no significant difference (*p* > 0.05). Cement leakage was found in 3 cases (3.85%) in Group A and 1 case (4.17%) in Group B, with no statistical difference (*p* > 0.05). None of the above 4 patients with cement leakage had serious symptoms. During follow-up, there were 2 cases (2.56%) of fractured vertebrae recollapse in Group A, with no obvious pain symptoms. Adjacent segment fractures were found in 5 cases (6.41%) in Group A and 1 case (4.17%) in Group B, with no statistical difference (*p* > 0.05). Six patients with adjacent segment fractures did not undergo surgery again due to no obvious pain symptoms and progressive kyphosis.

## Discussion

With the accelerated progress of aging society, OVCF, mainly caused by osteoporosis, has become an important health problem all over the world. In recent years, PKP has been widely used in the treatment of OVCF because it can obtain some benefits in short-term prognosis, including rapid pain relief, recovery of AVH and shortening bed rest time. Due to the risk of severe cement leakage and the difficulty of surgical techniques, some authors previously considered sOVCF as an absolute or relative contraindication for PVP (12, 21, 22). However, through the mastery and improvement of surgical techniques, more and more researchers have conducted studies on patients with sOVCF and confirmed that PKP is also effective for these patients (23–25). In a retrospective study conducted by Wen et al. (25), patients with sOVCF reported satisfactory improvements in VAS and ODI scores, LKA, and AVH after PKP compared with the pre-operative values (*p* < 0.05).

However, with the wide application of PKP and the deepening of related research, the complications caused by PKP have attracted increasingly attention, including cement leakage, fractured vertebrae recollapse and fractures of adjacent segments (26–29). Therefore, to give full play to the advantages of PKP and reduce the incidence of these complications, some studies have applied PPSF combined with KP or VP to treat patients with OVCF (15–17). Gu et al. (15) reported that 68 patients with single-level thoracolumbar OVCF underwent PPSF+VP. The results showed that, compared with the pre-operative values, VAS scores, Cobb angle and AVH were significantly improved, and PPSF+VP had obvious effects on preventing fractured vertebrae recollapse and adjacent segment fractures. In 2021, Huang et al. (16) conducted a retrospective study and concluded that for patients with osteoporotic thoracolumbar fractures, PPSF+KP can not only achieve favorable outcomes but also maintain longer correction and stronger support of the vertebrae compared with PKP. In this study, we retrospectively compared the effects of PKP and PPSF+KP on clinical function and radiographic outcomes in patients with single-level thoracolumbar sOVCF. By evaluating the clinical function and radiological parameters of the two groups, significant improvements were found post-operatively, 1 month after surgery and at the final follow-up compared with the pre-operative results, suggesting that PKP and PPSF+KP were all effective treatment options for patients with single-level thoracolumbar sOVCF. The two surgical methods significantly improved the prognosis of patients, which was consistent with the results of other studies mentioned above. The reason may be that both PKP and PPSF+KP can significantly restore AVH, to effectively improve the stability of the anterior and middle columns of the compression fracture vertebrae and partially restore the anterior support function. In addition, compared with Group A, most perioperative parameters of Group B showed a better side, and the post-operative VAS and ODI scores of Group B were also lower, suggesting that PKP may be better than PPSF+KP in the short-term effects after operation. These were because PPSF+KP was surely more complex compared with PKP and caused greater trauma than PKP, which may affect the early post-operative pain relief and functional recovery.

In recent years, the spinal sagittal imbalance caused by OVCF has attracted some researchers' attention. Sutipornpalangkul et al. (30) confirmed that patients with OVCF had anterior wedge deformity, which led to the progression of kyphosis and the forward movement of the center of gravity, and finally lead to spinal sagittal imbalance. LeHuec et al. (31) reported that patients with OVCF had poor global sagittal alignment and decreased quality of life, and the severity of vertebral compression fracture had a negative impact on global spinal sagittal balance. Furthermore, Cao et al. (32) found that OVCF in the thoracolumbar region had a greater impact on spino-pelvic alignment and global spinal sagittal balance than in other regions. PKP is an effective method for minimally invasive treatment of OVCF, but it is still controversial whether it is conducive to the recovery of global spinal sagittal balance (33–35). Kanayama et al. (33) and Sutipornpalangkul et al. (30) analyzed different numbers of OVCF patients treated with PKP and concluded that PKP was helpful for immediate pain relief, but did not improve the global spinal sagittal balance. However, some scholars have confirmed that PKP can improve spinal sagittal balance by restoring AVH and correcting LKA (32, 36). In our study, by evaluating the radiographic outcomes of both groups, including AVH, AVHr and LKA, PPSF+KP can more significantly restore AVH and AVHr, reduce LKA of the fractured vertebrae and increase LL after surgery than PKP. Furthermore, after more than 2 years of follow-up, AVH, AVHr and LKA, and some spino-pelvic sagittal balance parameters suggested that PPSF+KP may play a better role in maintaining spinal sagittal balance than PKP. Although few studies reported the effects of PPSF+KP on spino-pelvic sagittal balance in patients with sOVCF, through the discussion of other studies mentioned above, we can infer that the reasons for the differences between the two groups are as follows: On the one hand, PPSF+KP can effectively fix the upper and lower adjacent vertebral bodies of the fractured vertebral body, and exert a certain degree of traction on the compressed and fractured vertebral body, which can maximize the advantages of PKP in restoring AVH during the operation. This may also explain why PPSF+KP is better than PKP alone in the post-operative correction of LKA and maintenance of spinal sagittal balance. On the other hand, although PKP can also significantly restore AVH and correct LKA in the early stage, the loss of AVH and the aggravation of LKA are often caused by intravertebral cleft (37) and osteoporosis (38) with the passage of time. Therefore, without strong support of pedicle screw fixation, some patients may be at risk of spinal sagittal imbalance.

Spino-pelvic sagittal balance plays an important role in maintaining the normal physiological function of the spine, and normal spino-pelvic sagittal balance is crucial to maintain a stable posture and transfer normal axial stress (39). Pelvic parameters include PI, PT and SS. PT is a characteristic of pelvic rotation, and the standard value is about 13° ± 6° (40). Sung-Soo et al. (41) reported that patients with PT improvement showed significantly better VAS and ODI scores than those without improvement. In our study, there was a statistical difference in PT between the two groups at the final follow-up, which may explain why there were differences in ODI and VAS scores between the two groups. SS is defined as the angle between the horizontal line and the line parallel to the sacral plate, which is ~41° ± 8°. PI increases from age 4 to 18 but does not change further into adulthood (42, 43), and the standard value is ~53° ± 9° (44). PI, which is not affected by posture, can be used as an indicator to describe the shape of pelvis and sacrum orientation since the above three pelvic parameters fulfill the equation: PI = PT + SS (45). Changes in SS and PT can be viewed as changes to compensate for sagittal imbalance (36). LL is the angle between the superior endplate of L1 vertebrae and the sacral plate, and the standard value is ~46.5° (45, 46). There is a close relationship between LL and PI, and the ideal formula is: LL = PI ± 9°. If these two parameters do not match, it would cause the imbalance of spinal sagittal balance. Therefore, a new parameter, PI-LL, has been produced between PI and LL, which can more directly quantify the mismatch between pelvis shape and lumbar curve, so it can be used to guide the lumbar surgery plan and the recovery target of patients after surgery (47). One of the goals of spine pelvis sagittal alignment is that PI-LL <10° threshold (48). In this study, PI-LL of the two groups did not reach the ideal standard before surgery and improved significantly after surgery. Although there was no significant difference in PI-LL between the two groups, it was found that PI-LL of PPSF+KP was slightly lower than that of PKP during post-operative follow-up. Regarding the above results, the reason we infer is that these elderly osteoporotic patients have already a certain degree of spinal deformity before the vertebral fracture, and they often rest or lack daily activities after the operation. Therefore, even if two surgical methods are used to restore the height of the fractured vertebral body and correct the local kyphotic angle, they may have a limited effect on spinopelvic sagittal balance. However, these are only our current inferences, and more in-depth research and longer follow-up are needed to confirm these.

There have been some reports that secondary vertebral fractures after PVP or PKP, including further compression of previously treated vertebrae and new fractures in adjacent vertebrae (11, 16, 49, 50). Kim and Rhyu (49) showed that the incidence of fractured vertebrae recollapse was 12.5%. Lavelle and Cheney (50) found that the incidence of recurrent vertebral fractures after PKP was 10%. Rho et al. (11) reported that 27 (18.4%) of 147 patients treated with PVP or PKP subsequently developed new vertebral fractures, and 66.7% of 27 patients developed new fractures in adjacent vertebrae. In the PKP group of this study, 10 (12.82%) of 78 patients had complications, including cement leakage (*n* = 3), fractured vertebrae recollapse (*n* = 2) and adjacent vertebral fracture (*n* = 5), these incidences are slightly lower than the above-mentioned studies. In addition, Huang et al. (16) reported that 23 patients with osteoporotic thoracolumbar fractures (48.9%) in PKP group had complications, including cement leakage (*n* = 10), fractured vertebrae recollapse (*n* = 12) and reoperation due to refractures (*n* = 2), and the complications in PPSF+KP group were significantly less (*p* < 0.05), including cement leakage (*n* = 2), wound infection (*n* = 1), and recollapse at the final follow-up (*n* = 2). In this study, 3 (20.51%) of 24 patients had complications, including cement leakage (*n* = 1), fractured vertebrae recollapse (*n* = 1) and adjacent segment fracture (*n* = 1), and there was no significant difference in the incidence of each complication between the two groups. The above results may be due to the difference in the numbers of patients between the two groups, leading to a certain degree of statistical bias in the incidence of complications. Therefore, we cannot arbitrarily conclude that there is no difference in complications between the two surgical methods.

This study had several limitations. First, it was designed as a retrospective comparative study, and the sample size was relatively insufficient, especially in patients with PPSF+KP. The difference in the number of patients between the two groups may cause high statistical biases in some data. Second, this study did not study deeply the risk factors that that affected the spinal sagittal balance parameters in both groups. Therefore, future studies may require a prospective randomized controlled study and a longer time to follow up more patients and further analyze the risk factors that affect the spine sagittal balance.

## Conclusions

For elderly patients with single-level thoracolumbar sOVCF, both PPSF+KP and PKP can not only achieve favorable outcomes, but also maintain the spinal sagittal balance well. Compared with PPSF+KP, PKP showed more significant advantages in the early post-operative period due to the simpler process and less trauma during operation. However, in the long-term follow-up, PPSF+KP showed a better clinical effect and may be better to maintain the spinal sagittal balance than PKP.

## Data Availability Statement

The raw data supporting the conclusions of this article will be made available by the authors, without undue reservation.

## Author Contributions

QZ: conceptualization, methodology, investigation, software, and writing—original draft. JZ: methodology, data curation, and investigation. HL: conceptualization, methodology, and writing—original draft. WH: methodology, data curation, and investigation. LD: data curation, validation, and writing—review and editing. XZ: data curation and writing—review and editing. HY: conceptualization, methodology, and writing—review and editing. TL: conceptualization, methodology, validation, writing—review and editing, and funding acquisition. All authors contributed to the article and approved the submitted version.

## Funding

This work was supported by the National Natural Science Foundation of China (82072476), the Natural Science Foundation of Jiangsu Province (BK20191173), Youth Science and technology project of rejuvenating health through science and education in Suzhou (KJXW2019010), and Bethune special fund for strengthening treatment of pathological spinal fracture (BKJP201702).

## Conflict of Interest

The authors declare that the research was conducted in the absence of any commercial or financial relationships that could be construed as a potential conflict of interest.

## Publisher's Note

All claims expressed in this article are solely those of the authors and do not necessarily represent those of their affiliated organizations, or those of the publisher, the editors and the reviewers. Any product that may be evaluated in this article, or claim that may be made by its manufacturer, is not guaranteed or endorsed by the publisher.
